# Dendritic mitoflash as a putative signal for stabilizing long-term synaptic plasticity

**DOI:** 10.1038/s41467-017-00043-3

**Published:** 2017-06-26

**Authors:** Zhong-Xiao Fu, Xiao Tan, Huaqiang Fang, Pak-Ming Lau, Xianhua Wang, Heping Cheng, Guo-Qiang Bi

**Affiliations:** 1 Hefei National Laboratory for Physical Sciences at the Microscale, University of Science and Technology of China, Hefei, 230027 China; 2 School of Life Sciences, University of Science and Technology of China, Hefei, 230027 China; 3 State Key Laboratory of Membrane Biology, Institute of Molecular Medicine, Peking-Tsinghua Center for Life Sciences, Peking University, Beijing, 100871 China; 4 Beijing Key Laboratory of Cardiometabolic Molecular Medicine, Institute of Molecular Medicine, Peking-Tsinghua Center for Life Sciences, Peking University, Beijing, 100871 China; 5 CAS Key Laboratory of Brain Function and Disease, University of Science and Technology of China, Hefei, 230027 China; 6 CAS Center for Excellence in Brain Science and Intelligence Technology, University of Science and Technology of China, Hefei, 230027 China; 7 Innovation Center for Cell Signaling Network, University of Science and Technology of China, Hefei, 230027 China

## Abstract

Mitochondrial flashes (mitoflashes) are recently discovered excitable mitochondrial events in many cell types. Here we investigate their occurrence in the context of structural long-term potentiation (sLTP) at hippocampal synapses. At dendritic spines stimulated by electric pulses, glycine, or targeted glutamate uncaging, induction of sLTP is associated with a phasic occurrence of local, quantized mitochondrial activity in the form of one or a few mitoflashes, over a 30-min window. Low-dose nigericin or photoactivation that elicits mitoflashes stabilizes otherwise short-term spine enlargement into sLTP. Meanwhile, scavengers of reactive oxygen species suppress mitoflashes while blocking sLTP. With targeted photoactivation of mitoflashes, we further show that the stabilization of sLTP is effective within the critical 30-min time-window and a spatial extent of ~2 μm, similar to that of local diffusive reactive oxygen species. These findings indicate a potential signaling role of dendritic mitochondria in synaptic plasticity, and provide new insights into the cellular function of mitoflashes.

## Abstract (web summary)

Mitoflashes are dynamic events in mitochondria, associated with depolarization and release of reactive oxygen species, and have been associated with several cellular functions. The authors now show that in neurons, dendritic mitoflashes are involved in structural postsynaptic changes during LTP.

## Introduction

Synaptic plasticity is regarded as the neuronal basis of learning and memory^1–3^. It involves a series of subcellular signals and structural changes, including Ca^2+^ influx, activation of protein kinases, reorganization of the cytoskeleton, as well as synthesis and translocation of proteins^4–7^. Two distinct temporal phases of plasticity have been characterized: an early phase that involves signaling and structural changes within seconds to minutes after induction, and a late phase that involves enduring changes lasting for tens of minutes to hours^8–10^, yet the key process linking the two phases are less well defined. Like many other cellular events, these synaptic processes are energy-consuming, and thus likely to require the involvement of mitochondria that play a pivotal role in the cellular and synaptic energy supply^11^. More than just a powerhouse, the mitochondrion is also emerging as an important signaling organelle that plays an active role in regulating synaptic Ca^2+^ signaling^12, 13^. In addition, mitochondrial dysfunction is known to result in synaptic plasticity defects and to be pivotal in various neurological disorders^12, 14, 15^.

Recently, it has been shown that respiring mitochondria exhibit a dynamic activity known as “mitochondrial flash” or “mitoflash”, a transient event comprising mitochondrial depolarization, reactive oxygen species (ROS) production, and alkalization in the matrix^16–21^. Mitoflash activity is closely associated with cellular and whole-animal metabolic state^22^, and modulates neural progenitor cell differentiation and proliferation^23^ as well as somatic cell reprogramming^24^. Acting as a digital reporter of the aging process, the frequency of mitoflash in a young adults can also predict the lifespan of *Caenorhabditis elegans* ^21^. Given that mitochondria constitute one of the most abundant organelles in neuronal processes, it is conceivable that such mitoflash events, if occurring at synapses, may also participate in activity-induced synaptic plasticity.

In the current study, we explore whether and how mitochondrial flashes (mitoflashes) participate in synaptic plasticity in cultured hippocampal neurons, a model system that has been used to identify important cellular mechanisms and computational rules of synaptic plasticity^25–28^. Specifically, we aim at determining whether activities that lead to functional and structural changes at the synapse can also cause mitoflash production in dendritic mitochondria, and, if so, whether such mitoflashes can reciprocally impact on the outcome of synaptic plasticity. Because of the heterogeneity of synaptic plasticity across individual synapses, we employ various stimulation paradigms used in previous studies of long-term potentiation (LTP) to induce lasting morphological enlargement of individual dendritic spines, a phenomenon termed structural long-term potentiation (sLTP)^29–32^. We find that induction of late-phase sLTP at synaptic spines is associated with a preceding increase in mitoflash frequency occurring in nearby dendritic shafts, whereas mitoflash activity remains unaltered during induction of only short-term spine enlargement. Artificially-eliciting mitoflashes stabilizes short-term spine enlargement and switches it into enduring sLTP. Furthermore, mitoflashes produce local ROS elevations on the micrometre scale, and ROS scavengers suppress mitoflash activity and impair late-phase sLTP expression, permitting only short-term spine enlargement. These results indicate that dendritic mitoflashes may be involved in short-term to long-term conversion of postsynaptic changes, and that may reflect a spatiotemporally specific, two-way communication between synaptic spines and dendritic mitochondria in hippocampal neurons.

## Results

### Dendritic mitoflashes in hippocampal neurons

**Fig. 1:**
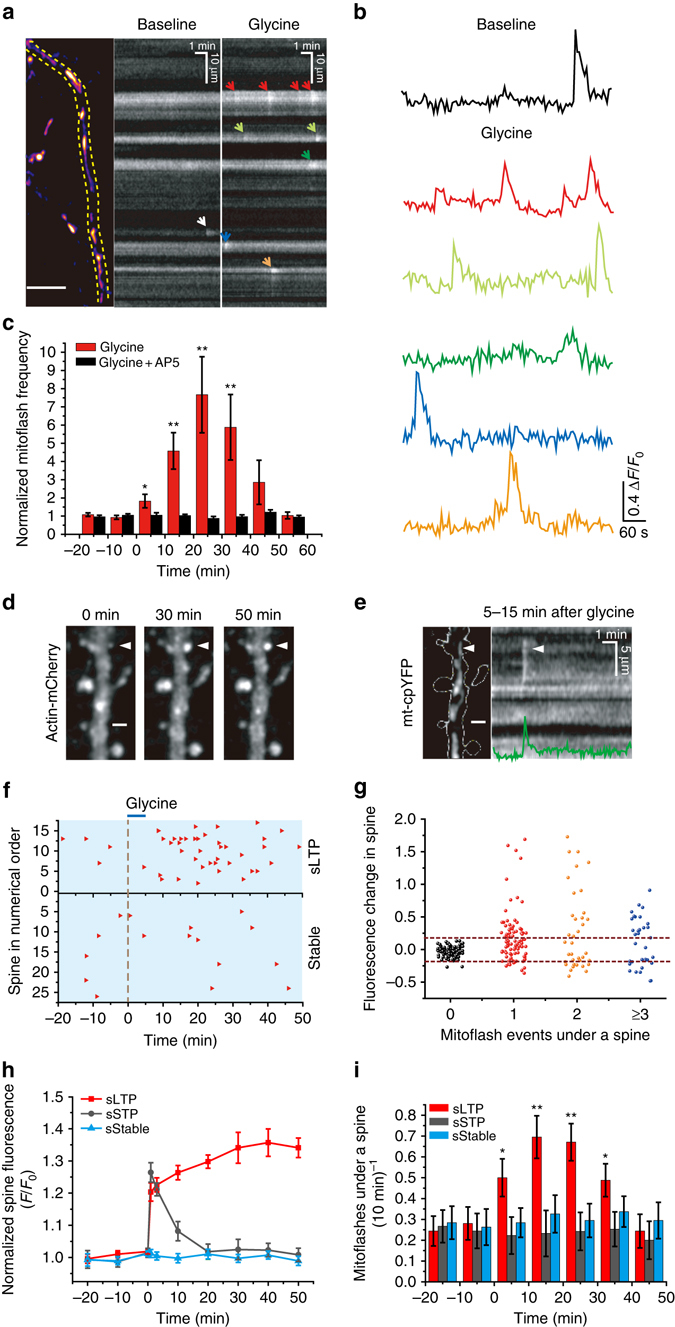
Dendritic mitoflashes occurring in chemical and electrical LTP induction. **a** Dendritic mitoflash activity before and after glycine stimulation. *Left*: mt-cpYFP fluorescence revealing mitochondria in a dendritic segment. *Dashed lines* delimit the boundaries of the segment. Scale bar, 10 μm. *Middle* and *right*: kymographs showing discrete mitoflashes (marked by *arrows*) prior to and 20–29 min after glycine treatment. **b** Time-courses of mitoflashes shown in **a**. **c** Phasic increase in mitoflash frequency after glycine stimulation (100 μM applied at time zero for 5 min). Note that this increase was abolished by D-AP5. *n* = 10–12 neurons from seven batches for each group. Unless otherwise stated, *error bars* in all figures hereafter report s.e.m. *P < 0.05, **P < 0.01 (paired *t*-test, after glycine vs. baseline). **d** Dendritic segment visualized by actin-mCherry at 0, 30, and 50 min after glycine treatment. Images are maximal projections of corresponding Z-stacks. *White arrowheads* mark a spine undergoing sLTP. Scale bar, 2 μm. **e** Mitoflash activity in the same dendrite as in **d**. *Left*: morphology of dendritic shaft and spines outlined by *dashed lines* overlaid on an mt-cpYFP fluorescence image. Scale bar, 2 μm. *Right*: kymograph and time-course (*green*) of a mitoflash event at the base of the marked spine in **d**, occurring at ~8 min after glycine stimulation. **f** Raster plot of mitoflash occurrence (*red triangles*) beneath 44 spines located on seven dendritic branches of a neuron, bisected into sLTP and stable groups according to size-changes after glycine stimulation (*blue bar*). **g** Relationship between spine size-change and the number of corresponding mitoflash events occurring 0–50 min after glycine treatment. Size-changes were quantified as mean actin-mCherry fluorescence intensity at 30, 40, and 50 min, normalized to that prior to stimulation. *Dashed lines* mark 2 SD of the fluctuation under basal conditions. *n* = 9 neurons from seven batches. **h** Long-term spine morphological changes induced by high frequency field electrical stimulation. *n* = 179 spines in eight neurons from four batches. **i** Mitoflash activity in dendritic mitochondria corresponding to the spines studied in **h**. *P < 0.05, **P < 0.01 (paired *t*-test, after electrical stimulation vs. baseline)

To explore the potential involvement of mitoflashes in synaptic plasticity, we used cultured hippocampal neurons^33^ transfected with mitochondrial matrix-targeting circularly-permuted yellow fluorescent protein (mt-cpYFP) as a mitoflash biosensor^16^. In the spiny dendrites of these neurons, mitochondria of various lengths occupied dendritic shafts in single file (Fig. 1a and Supplementary Fig. 1), and in the vast majority of cases, each spine had a mitochondrion at its dendritic base (>90%) or was within 2 µm of the nearest mitochondrion (99%) (Supplementary Fig. 1g, h). These dendritic mitochondria remained largely stationary during the period of observation, in contrast to frequent movements of axonal mitochondria, which were much smaller in size (Supplementary Fig. 2). Importantly, dendritic mitochondria underwent spontaneous mitoflash activity: individual events occurred as sudden increases of mt-cpYFP fluorescence intensity, lasted for tens of seconds, and were confined to single organelles (Fig. 1a, b; Supplementary Movie 1). Parallel multiparametric measurements revealed that mt-cpYFP-reported mitoflash activity was accompanied by alkalinization reported by mt-pHTomato (Supplementary Fig. 3a–c). The rising phase of the mitoflash was coupled with a step-increase of mitoSOX signal (Supplementary Fig. 3d), with mitoSOX irreversibly reacts with various ROS and displays a relative selectivity for superoxide. Further, concurrent mitochondrial depolarization was visualized as a decreasing tetramethylrhodamine ethyl ester (TMRE) signal (Supplementary Fig. 3e). Thus, dendritic mitoflashes of hippocampal neurons each consists of a ROS burst, a transient pH rise, and a reversible depolarization, and thus reflects an electrical and chemical excitation at the single-organelle level, as is the case in other cell types^16, 18, 34^.

### Dendritic mitoflashes accompanying sLTP at adjacent spines

To induce synaptic plasticity, we first used a chemical LTP (cLTP) induction paradigm^25^, which involved 5-min exposure to 100 µM glycine and resulted in a long-term increase in the amplitude of miniature excitatory postsynaptic currents (Supplementary Fig. 4). We found that a prominent phasic increase in mitoflash frequency peaked at ~20 min after glycine exposure (Fig. 1a–c). This increase was completely blocked by the N-methyl-D-aspartate receptor (NMDAR) antagonist D-AP5 (Fig. 1c), indicating that the glycine-induced mitoflash activity requires early Ca^2+^ influx through NMDARs, as is the case with the induction of cLTP^25^ as well as other forms of LTP^4, 27, 35^.

Using co-transfection with actin-mCherry and mt-cpYFP, we were able to monitor the structural changes of dendritic spines together with mitoflash events through the entire process of cLTP induction. Consistent with previous studies of cLTP and electrically-induced LTP^25, 36^, significant long-term morphological growth (termed structural LTP or sLTP hereafter) was observed in a subpopulation of dendritic spines (~30%) after glycine exposure, as evidenced by an increase of integrated actin-mCherry fluorescence that peaked early and persisted for at least 50 min after glycine stimulation (Fig. 1d). Interestingly, this subpopulation of spines, but not those that remained stable (~57%), exhibited higher dendritic mitoflash activity during the 10–40 min period following glycine exposure (Fig. 1e, f). Careful examination further revealed that all spines that underwent sLTP were each coupled with one or a few local mitoflashes during this period (Fig. 1f, g). It was also notable that ~13% spines decreased significantly in size following glycine treatment, and mitochondria adjacent to these spines also showed an elevated mitoflash frequency (Fig. 1g). Such spines might have undergone some form of long-term depression, in part influenced by sLTP in neighboring spines through heterosynaptic signaling^37^. These results suggest that dendritic mitoflashes may occur in the context of structural plasticity at dendritic spines.

In addition to chemical stimulation, we further employed the classical high frequency electrical field stimulation (HFS, 3 × 100 pulses at 100 Hz, 20 s apart) (Supplementary Fig. 5a) to induce sLTP. We found that after HFS, out of 179 spines examined, 62 (34.6%) spines showed long-term enlargement, 25 (14%) spines showed only short-term enlargement, and 92 (51.4%) spines were stable (Fig. 1h and Supplementary Fig. 12a). Again, only spines underwent long-term enlargement were found to have increased local dendritic mitoflash frequency within a 30-min time window after HFS (Fig. 1i and Supplementary Fig. 5b). These results indicate that spine sLTP is generally associated with a phasic increase in local dendritic mitoflash frequency.

**Fig. 2:**
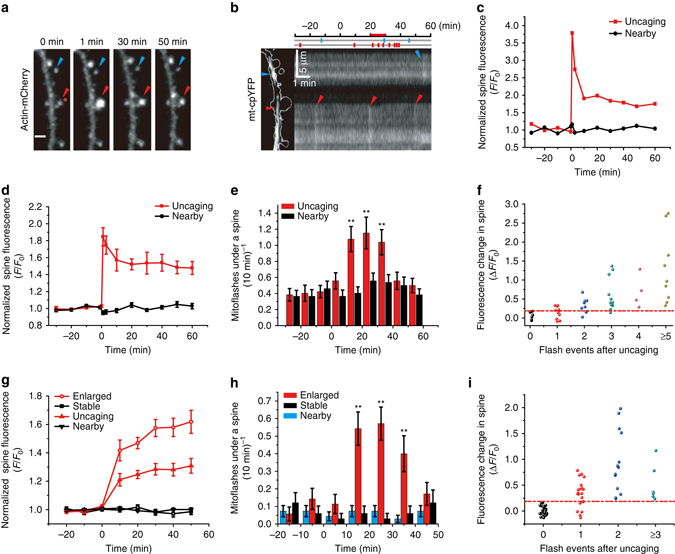
Dendritic mitoflashes occurring in sLTP induced by single-spine-targeted glutamate uncaging. **a** Sequential view of spine enlargement following the “HS” two-photon uncaging paradigm (720 nm at 8 mW, 120 pulses of 2.5 ms at 2 Hz, with 6 mM MNI-caged L-glutamate. See also Supplementary Fig. 6a). *Red dot* (at 0 min) denotes the uncaging spot placed close to a spine. *Red* and *blue arrowheads* mark this target spine and a nearby spine, respectively. Scale bar, 2 μm. **b** Local mitoflash activity beneath the spines. *Top inset*: raster plot of mitoflashes facing the target spine (*red ticks*) and a nearby spine (*blue tick*) during a 90-min period. *Left*: *White lines* outline the dendritic shaft and spines overlaid on the mt-cpYFP image of dendritic mitochondria. *Right*: kymograph showing mitoflashes between 20 and 30 min after glutamate uncaging. **c** Changes in actin-mCherry fluorescence of the target and nearby spines. **d** Statistics of sLTP induced in the HS paradigm. *n* = 51 spines in ten neurons from four batches for each group. **e** Time-dependent changes of mitoflash frequency corresponding to **d** (**P < 0.01; paired *t*-test). **f** Relationship between mitoflash number and spine size change. Data from the same experiments as in **d** and **e**. **g**–**i** Relationship between sLTP at individual spines and local mitoflash events, as in **d**–**f**, except that the LS paradigm was used (see also Supplementary Fig. 6a). *n* = 67 for target and nearby spines in 18 neurons from six batches. The target spines were further divided into responding (open circles, size change >2 SD of basal fluctuation, *n* = 33 spines in 15 neurons from six batches) and non-responding groups (*open squares*, *n* = 34 spines in 18 neurons from six batches). Note that no change in mitoflash frequency was found in non-responding spines (**P < 0.01; paired *t*-test)

In order to precisely delineate the relationship between synaptic plasticity and mitoflashes, we then targeted individual spines using two-photon photolysis of MNI-caged glutamate (4-methoxy-7-nitroindolinyl-caged-L-glutamate), similar to the method used to induce sLTP in hippocampal slices^38, 39^. Three paradigms of stimulation were created by varying MNI-caged glutamate concentration and the number of uncaging laser pulses (Supplementary Fig. 6a). In the high concentration, strong uncaging (HS) paradigm, a train of 120 two-photon pulses (720 nm, 2.5 ms, 8 mW) at 2 Hz in the presence of 6-mM MNI-caged glutamate induced sLTP at targeted spines, but not at neighboring spines (Fig. 2a, c), in agreement with previous reports^8, 40^. Kinetic analysis revealed a biphasic change of spine size, characterized by an early peak followed by a gradual ~16% decay, before stabilization at an elevated plateau. In a representative example, simultaneous mitoflash imaging detected seven events occurring in the mitochondrion immediately beneath the stimulated spine during the 10–40 min period after uncaging, but only one event in a nearby mitochondrion during the same period (Fig. 2b). Overall, during the 10–40 min after glutamate uncaging, a robust transient increase of mitoflash events occurred in the mitochondria underlying stimulated spines but not in those beneath nearby, unstimulated spines (Fig. 2e), the latter did not undergo sLTP (Fig. 2d and Supplementary Fig. 12b). It is noteworthy that no change in the amplitude or kinetics of mitoflashes was found after uncaging stimulation (Supplementary Fig. 6b–d). This result indicates that spine activity-triggered mitoflashes and spontaneous ones are indistinguishable, consistent with the notion that mitoflash, if serving a signaling role, would mainly operates via frequency-modulation^21, 22^.

With a lower concentration (2 mM) of MNI-caged glutamate, strong single spine-targeted glutamate uncaging (low-concentration strong stimulation, LS paradigm) (Supplementary Fig. 6a) elicited a slowly developing sLTP that exhibited a slow monophasic enlargement in 49% of spines (Fig. 2g and Supplementary Fig. 12c). An increase in mitoflash frequency was again seen only in mitochondria next to the enlarged spines ~10–40 min after uncaging, but not in those adjacent to stable spines (Fig. 2h). Moreover, in both the HS and LS paradigms, the number of mitoflash events beneath a stimulated spine appeared to positively correlate with the degree of spine size enlargement (Fig. 2f, i). These set of experiments illustrates a tight spatiotemporal specificity between sLTP at single spines and mitoflash activation in dendritic shafts.

**Fig. 3:**
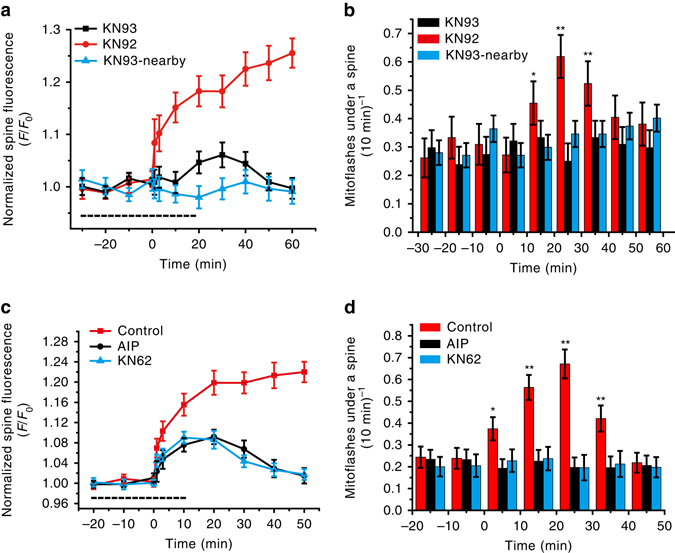
Dendritic mitoflashes occurring in sLTP depends on CaMKII activation. **a** Effects of the CaMKII inhibitor KN93 (10 μM) and its analog KN92 (10 μM) on sLTP induced by glutamate uncaging in the LS paradigm (see Supplementary Fig. 6a). *Dashed line* indicates the presence of KN93 or KN92. *n* = 6 neurons in four batches for the KN92 and KN93 groups. **b** Glutamate-uncaging-induced mitoflash frequency changes corresponding to the data in **a**. *P < 0.05; **P < 0.01; paired *t*-test vs. baseline. **c** Effects of the CaMKII inhibitors AIP (10 μM) and KN62 (10 μM) on sLTP induced by glutamate uncaging in the LS paradigm. *n* = 6 neurons in three batches for AIP groups, *n* = 5 neurons in three batches for KN62 groups, *n* = 7 neurons in three batches for control groups. *Dashed line* indicates the presence of AIP or KN62. **d** Glutamate uncaging-induced mitoflash activity was blocked by AIP and KN62. Data correspond to those in **c**. *P < 0.05; **P < 0.01; paired *t*-test vs. baseline

The induction and expression of sLTP at the dendritic spine involve a complex signaling cascade including the early processes such as Ca^2+^ ion influx through NMDA receptors and activation of Ca^2+^/calmodulin-dependent protein kinase II (CaMKII) and small GTPase^6, 41, 42^. The blocking effect of D-AP5 already showed an essential role of Ca^2+^ influx through NMDARs. However, since mitoflash activation peaked at ~20–30 min after spine stimulation, it appeared unlikely that Ca^2+^ transients directly trigger the delayed mitoflash response. To evaluate the role of signaling events downstream of Ca^2+^, we performed the same uncaging experiment in the presence of KN93, an inhibitor of CaMKII that is known to be crucial for LTP in hippocampal cultures as well as in other systems^28, 35^. Our results showed that both sLTP and sLTP-linked mitoflash activation were blocked by KN93, but not by the control compound KN92 (Fig. 3a, b). Similar results were obtained with two other CaMKII inhibitors, KN62 and autocamtide-2-related inhibitory peptide (AIP) (Fig. 3c, d). Thus, CaMKII activation as a key step in LTP induction is required for mitoflash activation.

### Mitoflashes during short- to long-term transition of sLTP

**Fig. 4:**
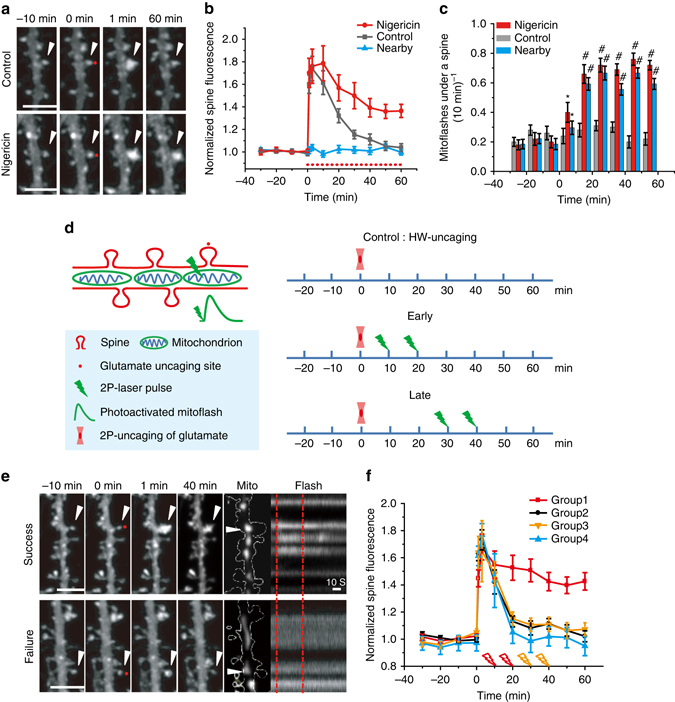
Artificially-eliciting dendritic mitoflashes converts short-term spine enlargement into sLTP. **a**, **b** Short-term spine enlargement induced by local glutamate uncaging using the HW paradigm (see also Supplementary Fig. 6a) and its conversion to sLTP by nigericin (50 nM). Representative examples and statistics are shown in **a** and **b**, respectively. *Arrowheads* in **a** denote uncaging target spines and *dotted line* in **b** the period of nigericin application. Note that nigericin did not alter the magnitude of enlargement at target spines and the size of nearby spines. *Red dots* (at 0 min) denote the uncaging spots placed close to spines. Scale bars, 5 μm. For controls, *n* = 51 spines on nine dendrites in six neurons. For nigericin and nearby groups, *n* = 45 spines on eight dendrites in six neurons. **c** Dendritic mitoflash events corresponding to the spines in **b**. Note that nigericin evoked greater, sustained mitoflash activity. *P < 0.05, ^#^ *P* < 0.01; paired *t*-test compared to baseline. **d** Experimental protocol with photoactivated mitoflashes (PA-mitoflashes). **e** Representative examples showing that PA-mitoflashes stabilized otherwise short-term spine enlargement into sLTP (*upper row*), whereas laser pulses per se in failed attempts were ineffective (*lower row*). Horizontal *arrowheads* denote the target mitochondria for photoactivation. *Vertical dashed lines* mark the timing of laser pulses. Scale bars, 5 μm. **f** Statistics. Group 1: at least one PA-mitoflash evoked at 10 or 20 min; Group 2: no PA-mitoflash evoked; Group 3: at least one PA-mitoflash evoked at 30 or 40 min; Group 4: no laser stimulation. *n* = 13 neurons and ten batches for group 1 (61 spines) and group 2 (53 spines), and *n* = 5 neurons and five batches for group 3 (28 spines) and group 4 (30 spines)

The HS paradigm-induced sLTP might be considered as a two-step process, a fast, early-phase enlargement followed by a slowly developing stabilization into a sustained structural change. Plasticity-triggered mitoflashes were found in experimental paradigms when sLTP was fully expressed. To evaluate mitoflash response to the early phases of spine plasticity, we used a weak uncaging paradigm (40 pulses at 2 Hz, HW) (Supplementary Fig. 6a), which was shown to induce only short-term enlargement of targeted spines^8^. Indeed, while spines responded with a ~1.8-fold peak enlargement, comparable to those in the HS paradigm, they completely recovered in ~40 min (Fig. 4a, b and Supplementary Fig. 12d), indicating that such spine enlargement failed to stabilize. Surprisingly, mitochondria at the bases of targeted spines showed no significant increase in mitoflash frequency (Fig. 4c), in contrast to their responses in the HS and LS paradigms. Thus, although one cannot exclude the possibility that spine plasticity-triggered phasic mitoflashes are an epiphenomenon accompanying synaptic changes, it is worthwhile to evaluate the potential role of mitoflash as a putative signal to stabilize spine enlargement after initial induction and to support the short- to long-term transition of sLTP.

One way to directly test our hypothesis is to artificially activate mitoflashes within the critical time-window prior to spine restoration, i.e., 10–40 min after initial induction of spine enlargement. For this purpose, we first employed the mitoflash activator nigericin, a K^+^/H^+^ antiporter, which has recently been shown in muscle cells to enhance mitoflash activity without disturbing the mitochondrial membrane potential when applied at low concentrations^18, 43^. We found that 50 nM nigericin rapidly and persistently augmented mitoflash frequency among the whole population of dendritic mitochondria in hippocampal neurons (Fig. 4c). More importantly, combination of the HW paradigm of single spine activation with immediate nigericin application resulted in persistent sLTP alongside mitoflash activation, suggesting that the artificially-elicited mitoflashes might indeed contribute to the conversion of otherwise short-term spine enlargement into sLTP (Fig. 4a, b and Supplementary Fig. 12d).

Next, we modified a mitochondrial photo-excitation protocol^44^ (see Methods, and Supplementary Figs. 3f–j, 7) to elicit a photo-activated mitoflash (PA-mitoflash) at a precisely designated time and location. In this experimental setting, two trains of femtosecond laser pulses (80 MHz, 720 nm, 10 mW, 1 ms; delivered at a 30-s interval) were targeted to a designated mitochondrion. The success rate of PA-mitoflash activation was ~50% and cases of failure were used as experimental controls (Fig. 4e, f). In contrast to Shi et al. (80 MHz, 810 nm, 10–17 mW, 100 ms–3 s)^44^, our laser stimulation by itself did not induce any detectable changes in local ROS or calcium concentration (Supplementary Figs. 3j, 7, 9), nor did it cause any mitochondrial swelling or fragmentation regardless of mitoflash activation (Supplementary Fig. 7). These PA-mitoflashes, as detected by multiple reporters including TMRE and mitoSOX, were indistinguishable from spontaneous and nigericin-triggered mitoflashes by virtues of amplitude and duration (Supplementary Fig. 3 and Supplementary Table 1). Strikingly, when combined with one or more early PA-mitoflashes (at 10 and/or 20 min after uncaging stimulation) (Fig. 4d), the HW paradigm evoked robust sLTP (Fig. 4e, f and Supplementary Fig. 12e). In contrast, no such sLTP was induced if the same laser pulses failed to elicit a PA-mitoflash (Figs. 4e, f and Supplementary Fig. 12e), or if the PA-mitoflash events were elicited late (30 and/or 40 min after uncaging) when the recovery from the early-phase spine enlargement was essentially complete (Fig. 4f and Supplementary Fig. 12e). Thus, dendritic mitoflashes evoked within a critical time-window appear to be effective in stabilizing spine size and permitting the full expression of sLTP. These results are consistent with a signaling role for mitoflash in the short-term to long-term transition of sLTP.

### Local ROS signaling in a mitoflash

**Fig. 5:**
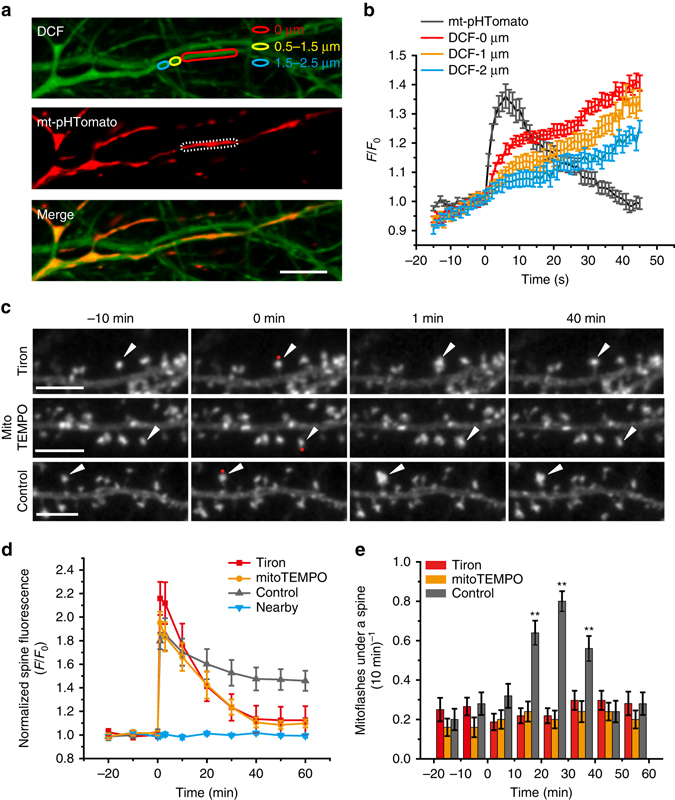
Local cytosolic ROS signaling during mitoflashes. **a**, **b** Representative images showing cytosolic DCF signal for ROS detection (*upper panel*), mt-pHTomato signal for mtioflash detection (*middle panel*), and their merged image (*bottom panel*). *Dashed line* in the *middle panel* delimits a spontaneously flashing mitochondrion, and local DCF signals were measured in regions of interest marked in the *upper panel*, at different distances from the mitoflash. Scale bar, 5 μm. **b** Time courses of local cytosolic DCF signals during mitoflashes. *n* = 72 events. See also Supplementary Fig. 8. **c** Representative examples showing inhibitory effects of the ROS scavengers, Tiron and mitoTEMPO, on sLTP induced in the HS paradigm. *Red dots* (at 0 min) denote the uncaging spots placed close to spines. *Arrowheads* denote uncaging target spines. Scale bars, 5 μm. **d** Time courses of sLTP induction under different conditions. *n* = 7 neurons and 4 batches for Tiron (45 spines), *n* = 7 neurons and four batches for mitoTEMPO (32 spines), and *n* = 7 neurons and four batches for control (48 spines) and nearby groups (48 spines). **e** Time courses of dendritic mitoflash frequency corresponding to **d**. The nearby group is omitted for clarity. **P < 0.01 vs. baseline

We hypothesized that diffusive messengers might emanate from the flashing mitochondria whereby dendritic mitoflashes drive the full induction of sLTP. We therefore measured local, cytosolic ROS (reported by 2',7'-dichlorofluorescein (DCF)), calcium (by GCaMP6f), and ATP (by magnesium green and PercevalHR) concentrations during mitoflashes. Our results showed that mitoflashes were accompanied by stepwise increases of cytosolic DCF signals that peaked in about 10 s and was spatially-graded over a distance of about 2 μm (Supplementary Fig. 8), indicating diffusion of mitoflash ROS into the cytosol (Fig. 5a, b). On the other hand, we detected no appreciable changes in local calcium and ATP concentrations during mitoflashes (Supplementary Figs. 9, 10). This does not argue against the importance of general ATP production in sLTP. In fact, inhibiting ATP synthesis with FCCP or other ETC inhibitors severely impaired spine actin dynamics and sLTP (Supplementary Fig. 11). However, it is unlikely that mitoflashes support sLTP as burst of ATP energy supply. Because recent studies have implicated ROS signaling in synaptic plasticity^45, 46^, we examined possible effects of the ROS scavengers Tiron and mitoTEMPO in glutamate uncaging-induced sLTP (Fig. 5c–e). Strikingly, both scavengers suppressed the phasic increase in mitoflash frequency in the HS paradigm (Fig. 5e), and effectively abolished sLTP, leaving only short-term spine enlargement (Fig. 5d and Supplementary Fig. 12f). These results are consistent with an involvement of mitoflash ROS in sLTP.

**Fig. 6:**
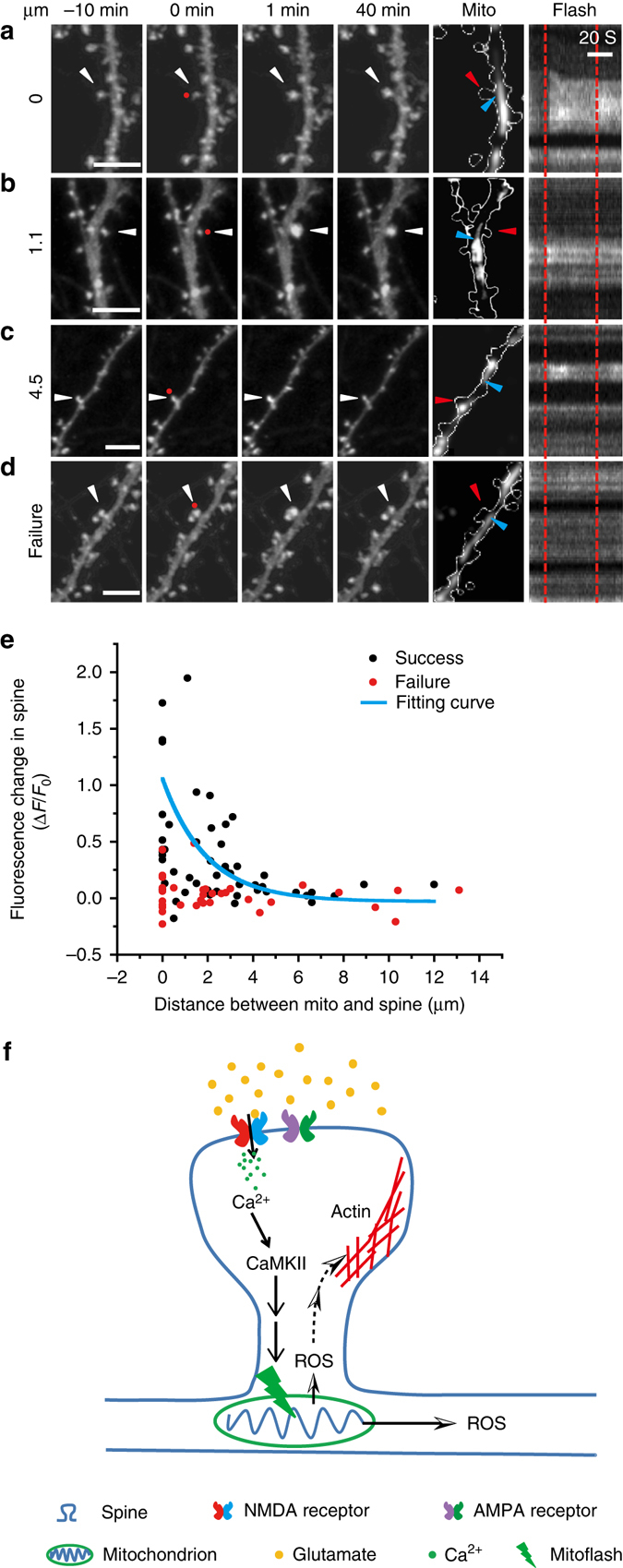
Spatial specificity of putative mitoflash signaling in sLTP at dendritic spines. **a**–**d** Representative examples of time-dependent spine enlargement with PA-mitoflashes elicited (or attempted) at various distances from the target spine 10 min after glutamate uncaging in the HW paradigm (See also Supplementary Fig. 6a). *Red dots* (at 0 min) denote the uncaging spots placed close to spines. *White arrowheads* denote uncaging target spines. *Red* and *blue arrowheads* mark target spines and target mitochondria, respectively. *Vertical dashed lines* mark the timing of laser pulses. Scale bars, 5 μm. **e** Degree of long-term change in spine size as a function of the spine-mitochondrion distance for successful PA-mitoflashes (*black dots*). A single-exponential curve fitting yielded a spatial decay constant of 1.94 μm. Cases with failed attempts are shown as controls (*red dots*). *n* = 12 neurons and ten batches for both the success (53 spines) and failure groups (34 spines). **f** Schematic summary of possible role of mitoflash signaling in the structural plasticity of dendritic spines. Activation of postsynaptic NMDARs causes Ca^2+^ influx and subsequent CaMKII activation in the spine, which leads to the generation of a local dendritic mitoflash via yet-to-be-delineated processes. Dendritic mitoflashes so activated stabilize spine enlargement likely via a diffusive ROS signal that targets the cytoskeleton

Further, using local PA-mitoflashes, we examined the spatial pattern of mitoflash signaling in sLTP. For this purpose, we elicited PA-mitoflashes at various distances from selected spines at 10 and 20 min after HW uncaging stimulation applied to the spines (Fig. 6a–d). We found that the mitoflash signaling in terms of stabilizing spine enlargement was highly confined to within only a few micrometres. Within this short distance, the farther away the PA-mitoflashes were, the less the spine growth could be sustained. Quantitatively, the degree of persistent spine enlargement fitted an exponential decay function of the distance between spine and PA-mitoflash, yielding a length constant of ~2 µm (Fig. 6e), similar to the range of cytosolic diffusion of mitoflash ROS as indicated by DCF signals. Although these observations do not exclude the possibility that the ROS elevation is an epiphephenomon of mitochondrial activity, they are consistent with the scenario that local cytosolic ROS transient in a mitoflash provides a signal for stabilizing structural plasticity at the spine.

## Discussion

We present two findings suggesting a previously unknown interplay between synaptic plasticity in the form of sLTP and dynamic activity of dendritic mitochondria in the form of mitoflashes (Fig. 6f). Firstly, early signaling events during sLTP induction in a spine cause a phasic increase in mitoflash frequency in adjacent dendritic mitochondria, regardless whether the induction is through glycine treatment or high-frequency electrical stimulation or single-spine-targeted focal glutamate uncaging. This finding provides direct evidence that dendritic mitochondria can act as an organelle biosensor capable of decoding the complex cellular signal originated from the spine. It is clear that key signals for LTP induction, including Ca^2+^ influx through NMDARs and subsequent CaMKII activation^2, 4, 6^, participate in this spine-to-mitochondrion signaling. Basal ROS signal is also involved because ROS scavengers effectively inhibit the sLTP-linked mitoflash activity. Albeit obligatory, the transient Ca^2+^ signal and CaMKII activation are unlikely the direct trigger, because the synaptic activity-linked mitoflashes occur within a broad 30 min window after synaptic stimulation. One possibility is that downstream processes responsible for spine enlargement, such as receptor recruitment and cytoskeletal reorganization, consume more energy and thereby alter metabolic state in nearby mitochondria. Indeed, it has been shown that, in other types of cells, mitoflash frequency varies depending on cellular metabolic state^22, 47^. Alternatively, the delayed mitoflash activation might be triggered by metabolically independent signals occurring late in the sLTP process.

Secondly, we found that dendritic mitoflashes appear to act reciprocally on the synapse, to stabilize spine enlargement and switch on the full expression of sLTP. Phasic increase of mitoflash activity is associated with full-fledged sLTP, but not when only a short-term enlargement is expressed, and this is true for all the chemical, electrical, and photolytic paradigms of synaptic stimulation. Manipulations that inhibit mitoflashes prevent sLTP without affecting the expression of short-term spine growth, suggesting that the mitoflash-sLTP link is causal and specific. Conversely, when combined with weak stimulations, artificially activating the mitoflash, with either chemical stimulation or photo-excitation, is sufficient to stabilize otherwise short-term spine enlargement into long-term sLTP. With precise photo-activation methods, we demonstrate that this mitochondrion-to-spine signaling displays high spatiotemporal specificity: PA-mitoflashes are effective in supporting sLTP only if they are evoked during the early time window after spine stimulation (<30 min) and within a distance of a few micrometres. These results suggest that, in addition to being a biosensor, the mitochondrion has the potential to act as a signaling organelle and play a role in the signaling cascade of synaptic plasticity in hippocampal neurons.

Alternatively, mitochondrial activity may play a permissive role in sLTP as the mitochondrion in close proximity to a spine undergoing sLTP are providing the necessary energy required for this process. In such a scenario, the mitoflashes could simply reflect bursts of energy production in response to cellular stress due to increased energy demand from the changing spine. However, measurements with both magnesium green and percevalHR failed to reveal any fluctuations in local ATP concentration during mitoflashes. In contrast, mitoflashes are accompanied by transient increases in local ROS, but not calcium, that spread over a few micrometres, in a spatial pattern closely resembling that of mitoflash regulation of sLTP. Therefore, while energy provision by the mitochondria is undoubtedly crucial for long-term synaptic modification, and we cannot fully exclude the possibility that mitoflash is an epiphenomenon during sLTP, it is possible that the role of mitoflashes go beyond bioenergetics. Specifically, we hypothesize that mitoflashes generate local ROS transients to signal for the stabilization of spine sLTP. Indeed, emerging evidence indicates that ROS can play physiological roles in various cellular processes such as regulating synaptic transmission^48^ and modification of the actin cytoskeleton via its interaction with small GTPases^34, 49^, and ROS may be directly involved in signaling synaptic plasticity^12, 42, 45^. While previous studies have focused on roles of extracellularly and cytosolically generated sustained ROS in LTP induction^45^, our work points to a new scenario in which bursts of mitochondria-generated ROS occur locally and timely to support the expression of sLTP in adjacent spines. Intriguingly, from the LTD-like size reduction and increase in mitoflash occurrence observed in a small portion of spines following glycine stimulation (Fig. 1g), it appears that the mitoflash and perhaps the resultant ROS transient also stabilizes structural changes in the negative direction. More generally, mitoflashes produce packets of ROS signal that are qualitatively distinguishable from basal mitochondrial ROS produced by constitutive electron leakage of the electron transfer chain. Whereas sustained elevation of global ROS is often detrimental, mitoflash-generated ROS can be event-driven, spatially confined, and promptly extinguished. All these salient features point to an appealing cellular mechanism for generating brief and intense ROS pulses, which could in turn activate high-threshold ROS effectors and pathways locally. Such a mechanism that exploits the handy mitochondrial ROS machinery and the ROS signaling toolkits could in principle exist in various cellular contexts and should be evaluated in future studies.

The induction and action of mitoflashes thus may add a novel, spatiotemporally specific mechanism to the picture of the complex and dynamic cellular signaling network underlying synaptic plasticity^6, 28^. As such, tuning mitoflash activity might afford an effective means to regulate structural plasticity at synapses. Furthermore, given that more than one spine can share an extended mitochondrion, this spine-mitochondrion communication might provide a basis for a subtle form of “inter-spine association” whereby one spine can facilitate sLTP in another. Such a process could contribute, together with other diffusible signaling mechanisms (e.g., Ca^2+^ and ATP)^8^, to the clustering of dendritic spines during learning^40, 50–52^. Further studies of the two-way signaling between synapses and mitochondria are likely to reveal new mechanistic insights into synaptic plasticity and mitochondrion-related brain disorders.

## Methods

### Isolation and culture of primary hippocampal neurons

All procedures were performed following the guidelines of the Animal Experiments Committees at the University of Science and Technology of China and Peking University.

Primary cultures were prepared using neurons from embryonic rat hippocampus^33^. Briefly, both hippocampi of randomly selected E18 embryos (without distinguishing sex difference) from timed-pregnant rats (Beijing Vital River Laboratory Animal Technology Co. Ltd., Beijing, China) were removed and digested with 0.25% trypsin (Sigma-Aldrich, St. Louis, MO) for 15 min at 37 °C, followed by washing in Hanks’ balanced salt solution (HBSS) without Ca^2+^ and Mg^2+^ (Thermo Fisher, Waltham, MA) and gentle pipetting. The dissociated neurons were then plated on poly-L-lysine (Sigma-Aldrich)-coated glass coverslips in 35-mm petri dishes at 1 × 10^5^ cells ml^−1^ and kept in serum-free medium (plating medium: Neurobasal medium (Thermo Fisher) supplemented with 2% B27 (Thermo Fisher), 0.5 mM L-glutaMAX (Thermo Fisher), 37.5 mM NaCl (Sigma-Aldrich), and 25 μM L-glutamic acid (Sigma-Aldrich)) in 5% CO_2_ at 37 °C. 4 days after plating, half of the plating medium was exchanged with new medium (Neurobasal medium supplemented with 2% B27, 0.5 mM L-glutaMAX, 37.5 mM NaCl). The medium was then replaced every 4 days.

### Plasmid transfection

Cultured neurons were transfected with an mt-cpYFP-containing plasmid (CaMKII-mt-cpYFP) and a βactin-mCherry-containing plasmid (CaMKII-βactin–mCherry) at 8–9 DIV (days in vitro) using a calcium phosphate transfection kit (Thermo Fisher). Briefly, neurons cultured on coverslips were transferred to a new petri dish with pre-warmed transfection medium (Neurobasal medium supplemented with 2% B27, 0.5 mM L-glutaMAX, 37.5 mM NaCl, pH 7.4), while the culture medium was preserved in the original dish. CaMKII-mt-cpYFP (7.5 μg) and CaMKII-βactin-mCherry DNA (2.0 μg) along with 15.5 μl of 2 mol l^−1^ CaCl_2_ were diluted in ddH_2_O (125 μl in total). The DNA solution was added into 125 μl 2× HBSS followed by slow pipetting and gentle vortexing with an eighth volume added each time. The mixture was then incubated for 15–20 min at room temperature and added dropwise to the culture dish with transfection medium. After incubating the neurons for 80 min in 5% CO_2_ at 37 °C, an evenly distributed layer of precipitate particles was observed across the neurons under a ×4 objective. The DNA–Ca^2+^-phosphate precipitates were dissolved with freshly-made dissolution medium (feeding medium with extra 20 mM HEPES, pH 6.8) and incubated for 4–8 min at room temperature. Typically, no precipitate particles were then observed. The transfected neurons were then transferred back to their original dishes containing culture medium. The same transfection protocol is used for all the other plasmids transfection. All Imaging experiments were done with 17–20 DIV neurons, except for Fig. 2g–i, which were done with 13–15 DIV neurons.

### Electrophysiology and cLTP induction

Whole-cell perforated patch recordings were performed on 13–15 DIV neurons, using amphotericin B for perforation. The glass micropipettes were made on a PC-10 puller (Narishige, Tokyo, Japan), and had resistances of 2–4 MΩ. The pipettes were tip-filled with internal solution [containing (in mM): potassium gluconate 136.5, KCl 17.5, NaCl 9, MgCl_2_ 1, HEPES 10, EGTA 0.2, pH 7.20] and then back-filled with internal solution containing 150 ng ml^−1^ amphotericin B. Neurons were perfused with fresh extracellular bath solution (ECS) [containing (in mM): NaCl 150, KCl 3, CaCl_2_ 3, HEPES 10, glucose 8, tetrodotoxin 0.0005, strychnine 0.001, and bicuculline methiodide 0.02, pH 7.30] at a slow rate (0.7 ml min^−1^) throughout recordings. Miniature synaptic currents were recorded with a patch-clamp amplifier (MultiClamp 700B; Molecular Devices, Sunnyvale, CA) on an inverted microscope (Carl Zeiss Axio Observer, Oberkochen, Germany) with phase optics. For cLTP induction, cultured hippocampal neurons were treated with glycine (100 μM) in ECS for 5 min and then transferred to ECS without glycine. Signals were filtered at 5 kHz and sampled at 10 kHz. Data were analyzed with Igor Pro (WaveMetrics, Portland, OR). All experiments were performed at room temperature.

### Field electrical stimulation for LTP induction

Primary hippocampal neurons were perfused in extracellular solution containing (in mM): NaCl 150, KCl 3, CaCl_2_ 3, HEPES 10, MgCl_2_ 2, glucose 8, pH 7.30, at the speed of 0.7 ml min^−1^. Electrical field stimulation of about 15 V cm^−1^ was applied through a pair of platinum wires, using 5 ms voltage pulses delivered from high current capacity stimulators. Neurons were stimulated with high frequency stimulation (HFS, 3 X 100 pulses at 100 Hz, 20 s apart) to induce LTP.

### Imaging mitoflashes and spine morphology

An upright laser-scanning confocal microscope (Zeiss LSM NLO 710, Oberkochen, Germany) equipped with a water-immersion objective (WPlan-Apochromat ×20, numerical aperture 1.0, Zeiss) or an inverted confocal microscope (Zeiss LSM NLO 710) equipped with an oil-immersion objective (Plan-Apochromat ×40, numerical aperture 1.3, Zeiss) was used for imaging. Neurons cultured on a coverslip were perfused with extracellular solution containing (in mM): NaCl 150, KCl 3, CaCl_2_ 3, HEPES 10, D-glucose 8, tetrodotoxin 0.0005, pH 7.4) at rate of 1 ml min^−1^ in a perfusion chamber. For cLTP induction, 1 μM strychnine and 20 μM bicuculline methiodide (Sigma Aldrich) were included in the extracellular solution.

Time-lapse images of mt-cpYFP were captured by excitation at 488 nm (laser power, 2–3%) and collecting the emission at 490–550 nm. In typical time-series recordings of mitoflashes, 350 frames of 512 × 512 pixels were collected every 10 min at 0.10–0.15 μm per pixel in bidirectional scanning mode, and the axial resolution was set to 0.8–1.0 μm. In glutamate-uncaging experiments, 270 frames were collected at 38 frames min^−1^ from 3 to 10 min after uncaging. For mitoSOX (Invitrogen) fluorescence measurement, the indicator (3 μM) was loaded at 37 °C for 20 min followed by three washes, and cells were imaged by excitation at 543 nm and collecting the emission at >585 nm.

We used ImageJ^53^, ZEN (Carl Zeiss), and MATLAB (MathWorks, Inc.) software for image analysis. For mitoflash detection, we analyzed the changes of mt-cpYFP fluorescence intensity in target dendritic mitochondria with Zeiss ZEN software. The standard deviation (SD) of *F*/*F*_0_ for the entire trace was calculated, where *F*_0_ is the average baseline fluorescence intensity. The criteria for a mitoflash event were: (1) peak F/F_0_ > 3 SD; and (2) full duration at half-maximum >3 s.

To analyze their structural plasticity, we measured changes in the intensity of actin-mCherry fluorescence in spines. Briefly, 3-D stacks of mCherry images were acquired by excitation at 543 nm and collecting the emission at >590 nm at a series of time points. Typically, 9–10 frames were collected at 0.10–0.12 μm per pixel in unidirectional scanning mode at axial intervals of 0.5–0.65 μm. One 3-D stack of mCherry images was acquired every 10 min, alternating with the mitoflash image acquisition described above. After X-Y drift correction with Image Stabilizer^54^, a time series of the maximal intensity projection was generated, and photobleaching correction was carried out using Bleach Correction^55^. Then, the fluorescence intensity of target spines was analyzed using ImageJ. The fluorescence intensity at different time points was normalized to the basal value (average fluorescence intensity before glycine or uncaging stimulation). The analysis of spine size was blinded to the results of nearby mitoflash events with one author analyzing spine size, and another analyzing mitoflash events separately. The dendrites under severe *Z*-axis drifts were excluded from analysis.

### ATP detection during mitoflashes

For simultaneous imaging of local ATP detection and mitoflashes, primary hippocampal neurons were either co-transfected with CaMKII-mt-pHTomato and CaMKII-PercevalHR or, in cells transfected with CaMKII-mt-pHTomato only, loaded with Magnesium Green, AM (ThemoFisher) prior to imaging, at 5 μM for 30 min and then washed for 3 × 5 min. Image acquisition was performed 7 days after transfection, by excitation at 488 and 543 nm and collecting the emission at 500–530 and >600 nm, respectively, for CaMKII-PercevalHR and CaMKII-mt-pHTomato co-imaging; by excitation at 488 and 543 nm and collecting the emission at 500–530 nm and >600 nm, respectively, for Magnesium Green and CaMKII-mt-pHTomato co-imaging.

### Dendritic ROS detection

The indicator DCF (Invitrogen, Carlsbad, CA) (5 μM) was loaded at 37 °C for 10 min followed by 3 × 5 min washes, and image acquisition was performed by excitation at 488 nm and collecting the emission at >500 nm when imaged alone or 500–530 nm when co-imaged with mt-pHTomato. To avoid mitochondrial enrichment of DCF, image acquisition was performed only within 30 min after washes.

### Calcium imaging

Neurons were transfected with CaMKII-GCaMP6f on 8–9DIV and used for imaging 1 week later. Image acquisition was performed by excitation at 488 nm and collecting the emission at >500 nm when imaging alone, or 500–530 nm when co-imaged with mt-pHTomato.

### Photo-activation of mitoflashes

Zeiss Multispot Macro was used to photoactivate mitoflashes. The protocol was modified from a previous method^44^ after optimizing the laser wavelength, power, train duration, and number of trains. Briefly, photo-illumination with a 720-nm laser (Ti: sapphire laser, 690–1080 nm, 80 MHz, 10 mW, Coherent, Santa Clara, CA) was positioned at a diffraction-limited spot on a mitochondrion, with train duration of 1 ms, train number of two, and train interval of 30 s. The probability at which single laser stimulation successfully induced a mitoflash was ~50%. In contrast to the previous work, our photo-activation protocol did not induce mitochondrial swelling and fragmentation (Supplementary Fig. 7), or local ROS or calcium change (Supplementary Figs. 7, 9).

### MNI-glutamate uncaging with the two-photon method

MNI-glutamate (Tocris, Bristol, UK) was photolyzed by a 720-nm beam at 8 mW from a Ti:sapphire laser (Coherent, 690–1080 nm). The stimulus duration was 2.5 ms and the photolysis procedure was controlled using a Zeiss Multispot Macro. We used three different photolysis paradigms. First, for strong uncaging with a high concentration of MNI-glutamate (HS-uncaging), 6 mM MNI-glutamate was used, and 120 episodes of laser illumination at 2 Hz were applied to a target spine. Second, for weak uncaging with a high concentration of MNI-glutamate (HW-uncaging), 6 mM MNI-glutamate was used, and 40 episodes at 2 Hz were applied. Third, for strong uncaging with a low concentration of MNI-glutamate (LS-uncaging), 2 mM MNI-glutamate was used, and 120 episodes at 2 Hz were applied.

### Statistics

Required sample sizes were estimated based on experience of similar experiments. The experiments were not randomized. Data are expressed as mean ± s.e.m. The paired *t*-test was used to determine the statistical differences between samples at different time points. The Mann–Whitney test was used to determine statistical differences between two groups. For paired *t*-tests, all data were drawn from normally distributed populations. For all statistical tests, tests were two-sided and a *P* value < 0.05 was considered statistically significant.

### Data availability

All supporting data are available from the authors upon request.

## Electronic supplementary material

Supplementary Movie 1

Supplementary Information
