# Supplementary material for: Dendritic mitoflash as a putative signal for stabilizing long-term synaptic plasticity
Source: Nat Commun. 2017 Jun 26;8:31. doi: 10.1038/s41467-017-00043-3 (PMC5484698; doi:10.1038/s41467-017-00043-3)
Supplement: Supplementary file 2 — Supplementary Information [file 41467_2017_43_MOESM2_ESM.pdf]

File Name: Supplementary Information
Description: Supplementary Figures and Supplementary Table

File Name: Supplementary Movie 1
Description: **Dendritic mitoflashes in a primary hippocampal neuron.** Dendritic mitochondria (green) and spines (red) are imaged in a DIV 18 neuron co-expressing mt-cpYFP and actin-mCherry. The movie contains 94 time points at 7.83s intervals. For each time point, the red channel is the maximal intensity projection of a z-stack of 6 mCherry images at 1-μm axial interval, whereas the green channel displays single mt-cpYFP image at a pre-selected focal plane. Arrowheads are added to indicate the onset of subsequent mitoflashes. Time-courses of these mitoflashes are simultaneously plotted on the right.

Supplementary Figures

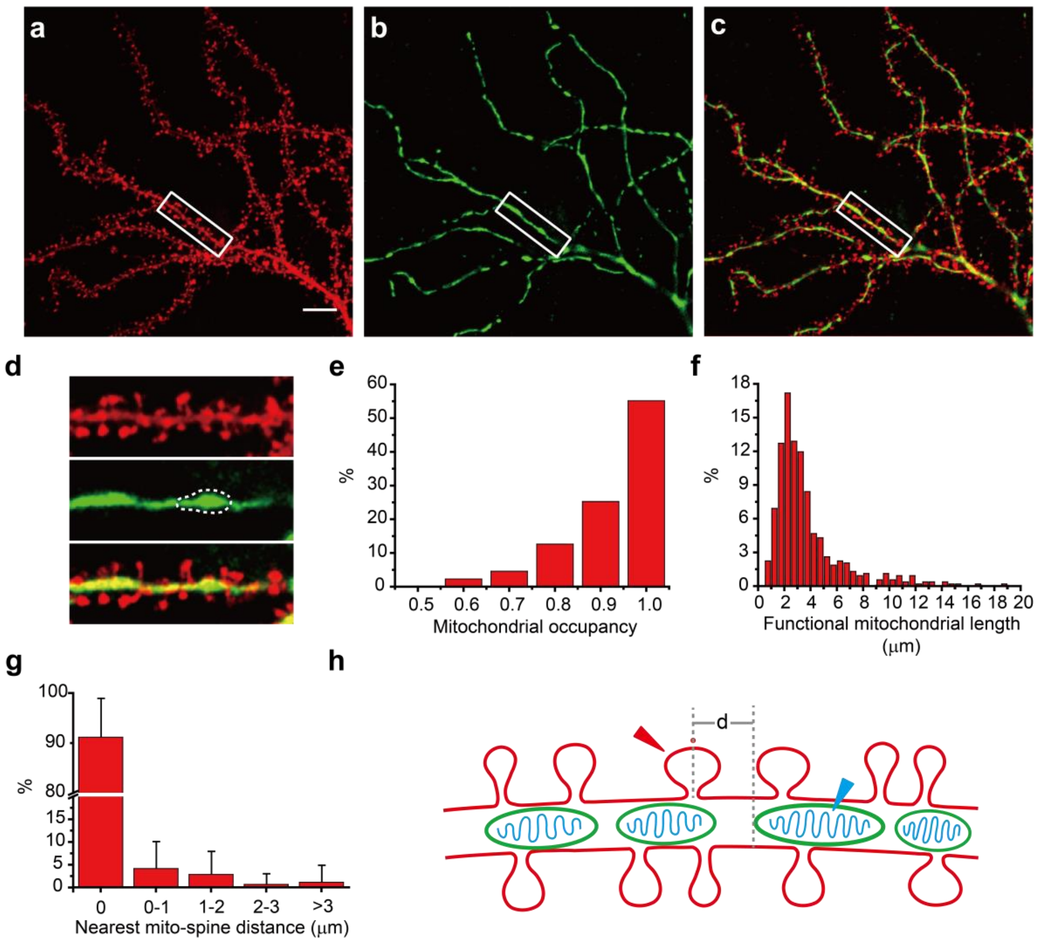

Supplementary Figure 1 Distribution of mitochondria in dendrites.

(**a-c**) Fluorescence images of dendrites (**a**) and dendritic mitochondria (**b**) visualized by actin-mCherry (**a**) and mt-cpYFP (**b**). Scale bar, 10 μm. (**c**) The merged image of (**a**) and (**b**). (**d**) Enlarged views of the boxed regions in (**a-c**). Dashed line outlines the boundary of a functional mitochondrion based on the spatial extent of a mitoflash. (**e**) Distribution of mitochondrial occupancy in dendrites. $Mitochondrial\ occupancy = \frac{dendritic\ mitochondrial\ length}{dendritic\ segment\ length}$. The dendrite between two adjacent branch points was considered to be a dendritic segment. n = 151 segments from 17 neurons. (**f**) Distribution of dendritic mitoflash length. n = 573 mitoflashes, the spatial extent of which reflect the length of functional mitochondria. (**g**) Distribution of the distance between a spine and its nearest mitochondrion in the parent dendrite (mean ± s.e.m.). Data are from 20 neurons. (**h**) Diagram defining the distance between target spine and stimulated mitochondrion. "d" is the distance between target spine neck and nearest edge of stimulated mitochondrion.

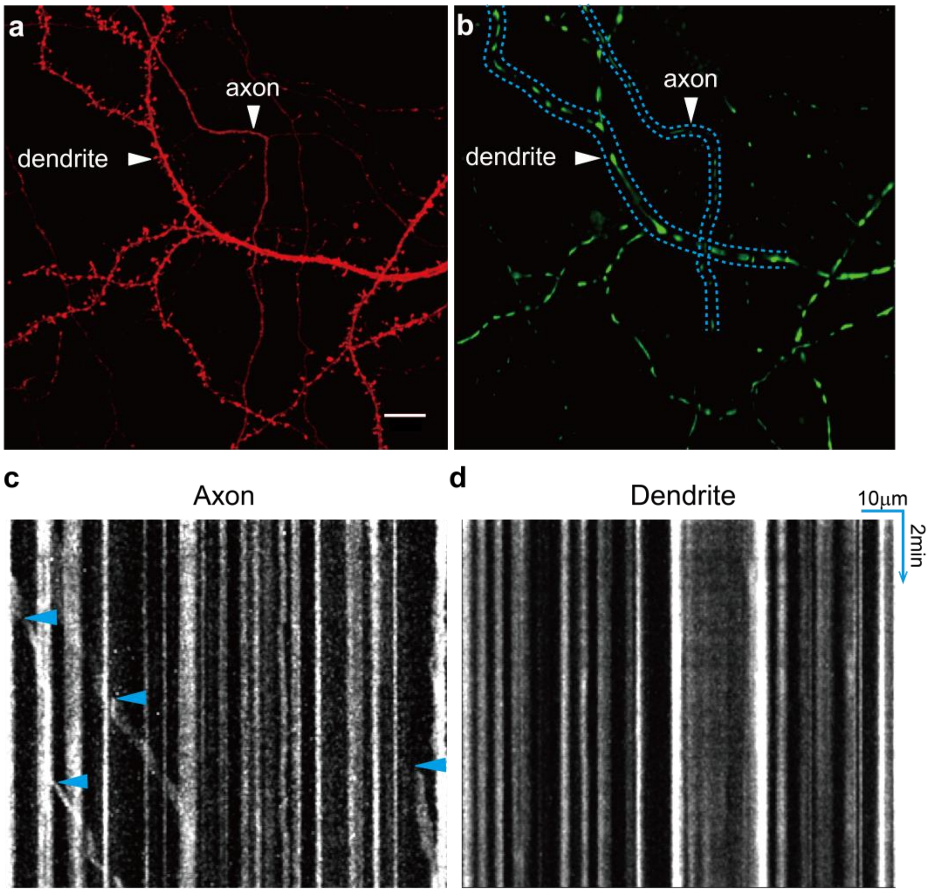

Supplementary Figure 2 Mitochondria are stationary in dendrites but mobile in axons.

(**a**) Dendrites and an axon visualized by actin-mCherry, Scale bar, 10 μm. (**b**) Mitochondria in the dendrites and axon visualized by mt-cpYFP. Dashed lines mark a 100-μm dendrite and a 100-μm axon. Note that axonal mitochondria are generally smaller and dimmer than those in dendrites. (**c**, **d**) Kymographs showing mitochondrial movement in an axon (**c**) and a dendrite (**d**) within 10 min. Blue triangles point to moving mitochondria.

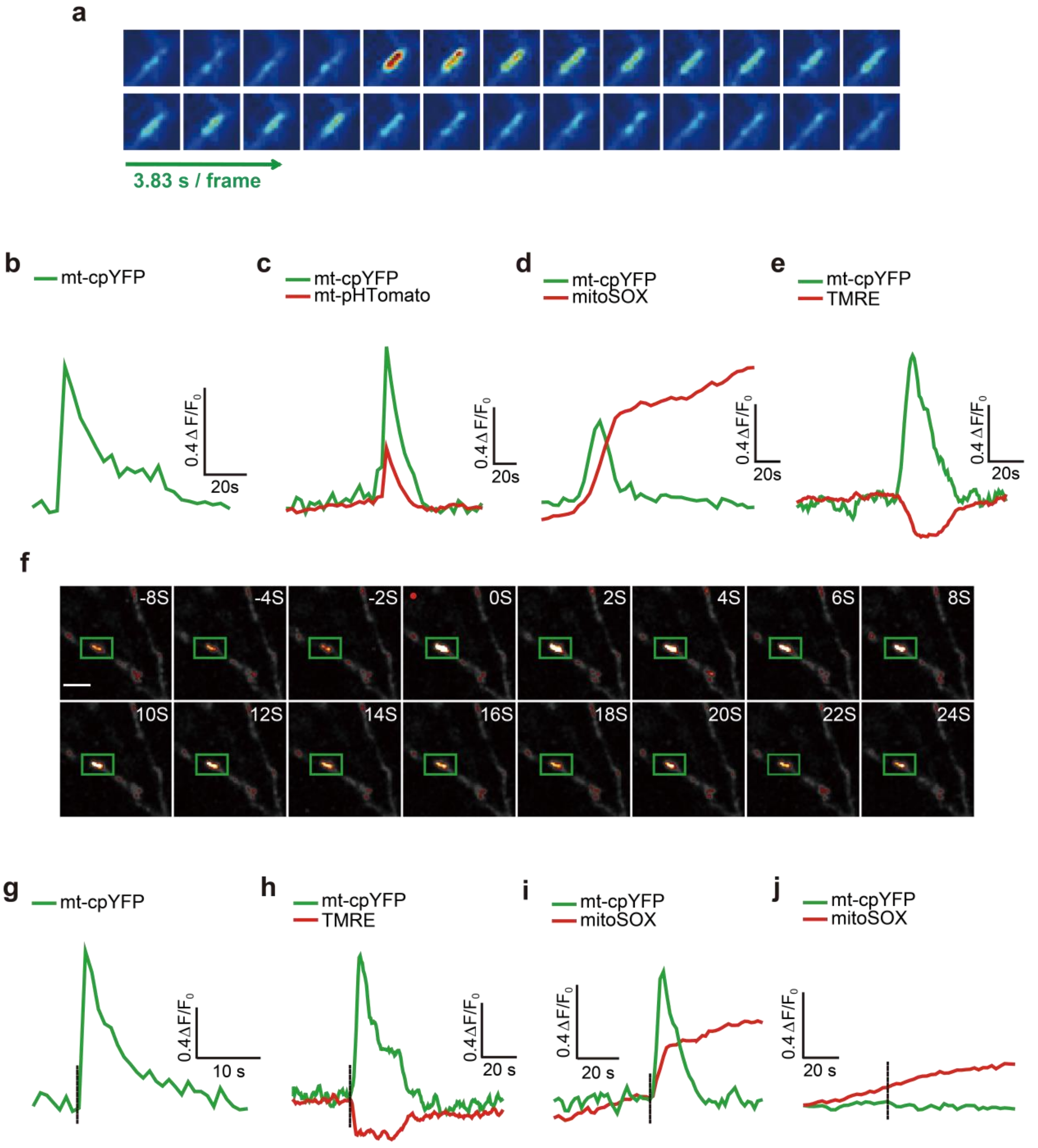

Supplementary Figure 3 Properties of spontaneous and PA-induced mitoflashes in hippocampal neurons.

(**a-e**) Properties of spontaneous mitoflashes in hippocampal neurons. (**a**) Time-lapse images of a dendritic mitoflash reported by mt-cpYFP and displayed at 3.83 s intervals. Horizontal side length = 5.4 μm. (**b**) Time course of the mitoflash in (**a**). (**c**) Mitoflash activity is accompanied by pH alkalization reported by mt-pHTomato, a mitochondrion-located pH-sensitive fluorescent protein. (**d**) The rising phase of the mitoflash is coupled with a step-increase of the mitoSOX signal. (**e**) Mitoflash is coupled with transient mitochondrial depolarization reported as a decreasing tetramethylrhodamine, ethyl ester (TMRE) signal. In all panels, similar results were obtained in at least 20 experiments. (**f-j**) Properties of PA-mitoflashes in hippocampal

neurons. (**f**) Time-lapse images of a PA-mitoflash. Green box: target mitochondrion for photoactivation. Red dot denotes the time point for photoactivation. Scale bar, 5 μm. (**g**) Time course of the PA-mitoflash in (**f**). Vertical dashed line: time of laser stimulation (720 nm at 10 mW for 1 ms). (**h**) PA-mitoflash is coupled with transient depolarization of the mitochondrial membrane potential as measured with TMRE. (**i**) PA-mitoflash is coupled with a stepwise increase of the mitoSOX signal. (**j**) Laser stimulation *per se* does not increase mitoSOX signal when it failes to elicit a PA-mitoflash. Vertical dashed line marks laser stimulation. See also (Supplementary Fig. 7).

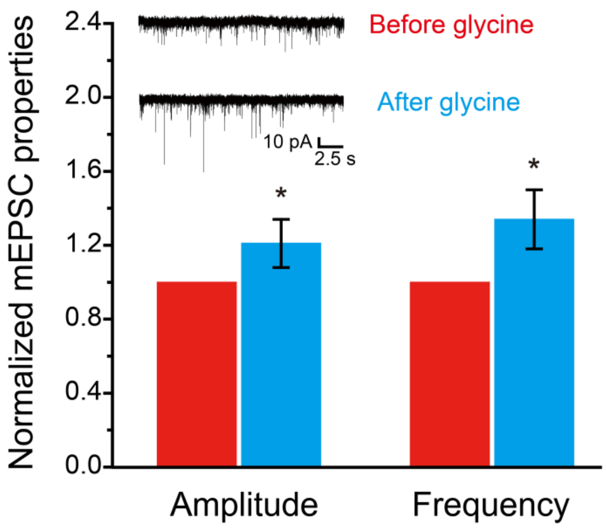

Supplementary Figure 4 Chemical LTP induced by glycine stimulation.

Bars show statistical analysis of changes in mEPSC amplitude and frequency. Insets are examples of electrophysiological recordings before and after 5 min stimulation with 100 μM glycine. Detailed experimental conditions are described in Methods. n = 9 neurons for 5 batches (results are presented as mean ± s.d.). *p <0.05; paird t-test vs baseline.

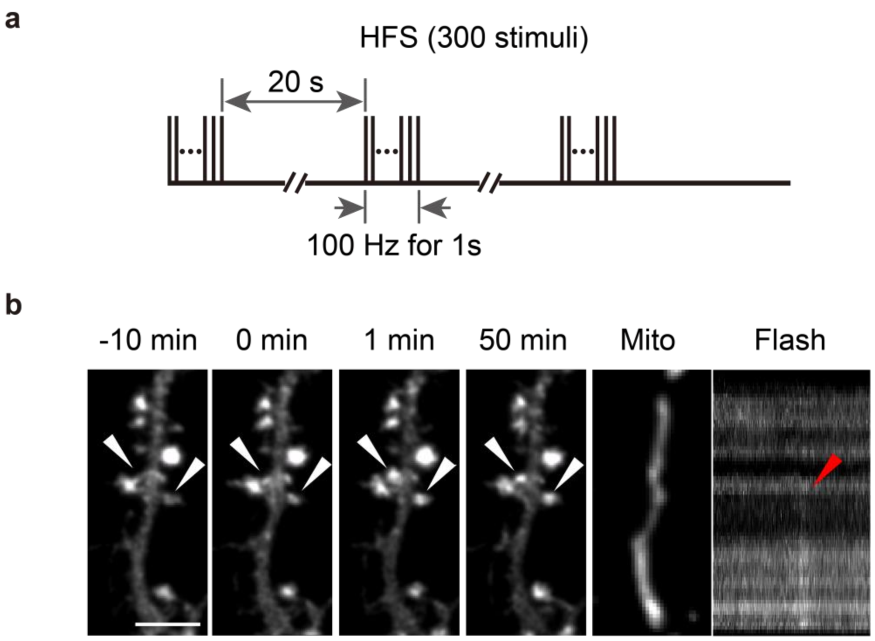

Supplementary Figure 5 Structural LTP induction by high frequency field stimulation.

(**a**) High frequency field stimulation (HFS) paradigm for induction of sLTP. 3 X 100 pulses at 100Hz, 20s apart. (**b**) Dendritic segment and mitochondria (Mito) were visualized by actin-mCherry and mt-cpYFP, respectively. Time-lapse images showing spine morphological changes following HFS. Rightmost kymograph was reconstructed along a line on the mitochondrion and corresponds to the period of 10-20min after HFS. White and red arrowheads mark, respectively, spines undergoing sLTP and a mitoflash occurring beneath them. Scale bar, 5 μm.

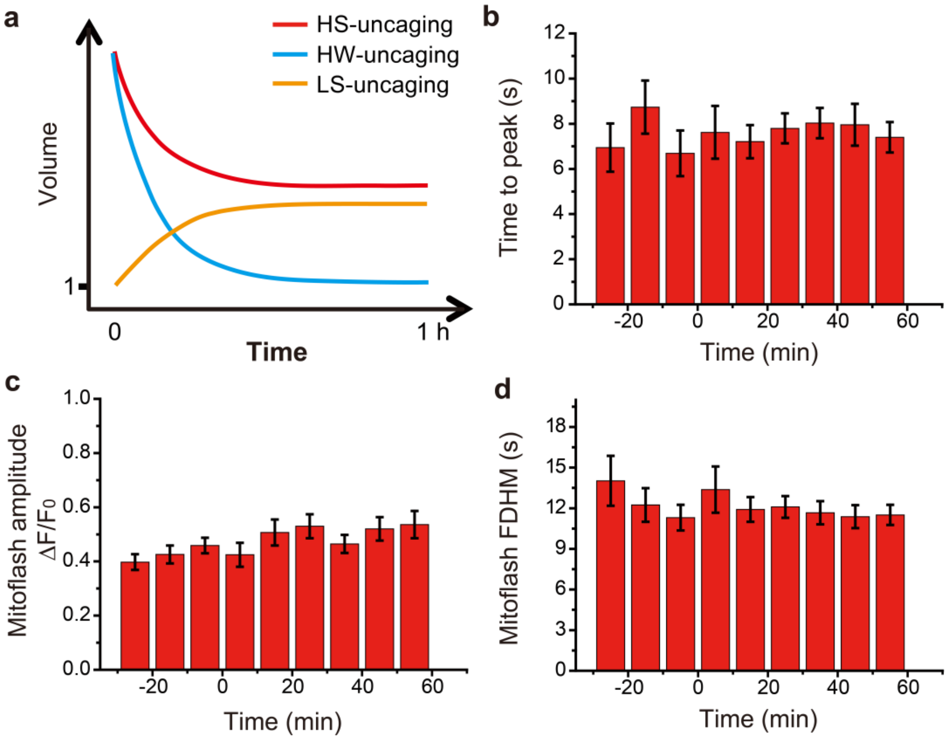

Supplementary Figure 6 Single-spine targeted glutamate uncaging induces CaMKII-dependent sLTP and phasic activation of mitoflashes.

(**a**) Schematic of the different glutamate-uncaging paradigms. Red, high-concentration strong glutamate-uncaging (HS-uncaging, 6 mM NMI-glutamate, 720 nm laser at 10 mW, 120 pulses, 2 Hz) induced both short-term (<30 min) and long-term spine enlargement. Blue, high-concentration weak stimulation (HW-uncaging, 6 mM NMI-glutamate, 720 nm laser at 10 mW, 40 pulses, 2 Hz) induced only short-term but not long-term spine enlargement. Yellow, low-concentration strong stimulation (LS-uncaging, 2 mM NMI-glutamate, 720 nm laser at 10 mW, 120 pulses, 2 Hz) induced long-term but not short-term spine enlargement. (**b-d**) Properties of individual mitoflashes remained unchanged before and after glutamate uncaging. Data correspond to the experiments reported in Figure **2e**. (**b**) Time-to-peak; (**c**) Amplitude; (**d**) Duration, indexed by full duration at half-maximum. n = 21-56 mitoflash events per bin. Error bars are SEM.

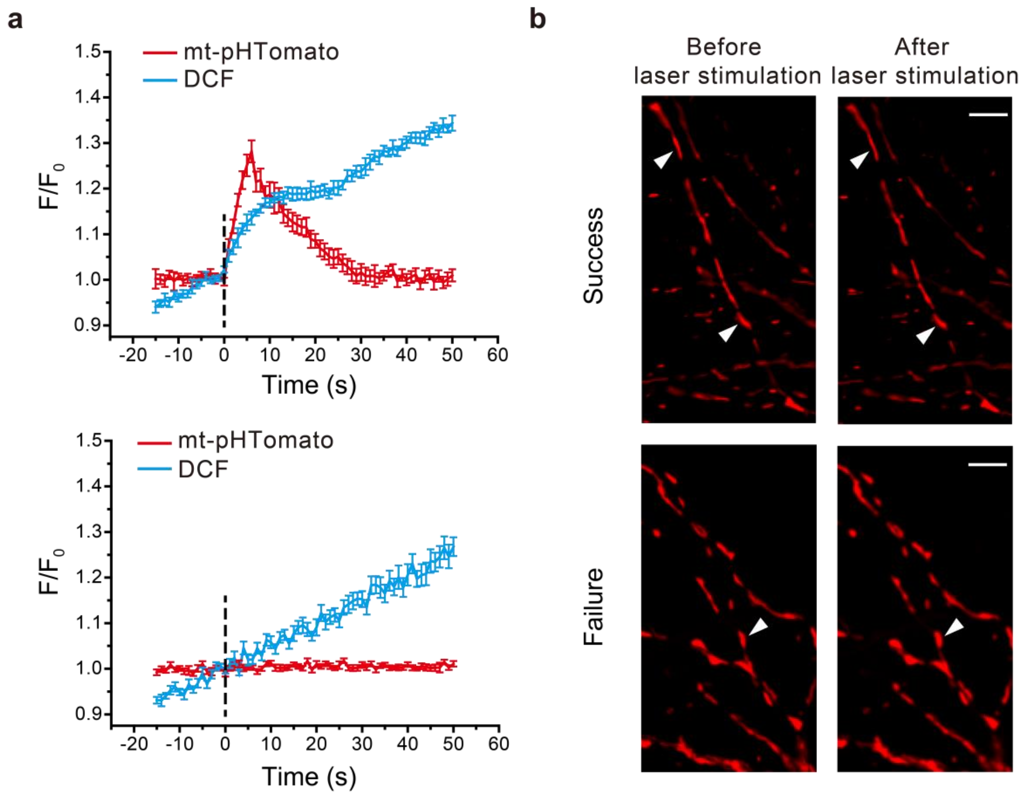

Supplementary Figure 7 Laser stimulation *per se* does not increase local ROS or impair mitochondria during the photoactivation of mitoflash.

(**a**) Local DCF signal change in response to laser stimulation with (upper panel) and without (lower panel) PA-mitoflashes (reported by mt-pHTomato) (mean±s.e.m.). Note that the DCF signals associated with PA-mitoflashes are almost identical to those of spontaneous mitoflashes (Fig **5b**). Note also the lack of any stepwise change in the DCF signal when same laser stimulation failed to activate a mitoflash. Vertical dashed line denotes the time of laser stimulation (720 nm, 10mW, 1ms). n= 54 successes and 59 failures. (**b**) Dendritic mitochondrial morphology (revealed by mt-pHTomato) before and after laser stimulation with and without mitoflash activation. White arrowheads mark target mitochondria. Scale bar, 5μm.

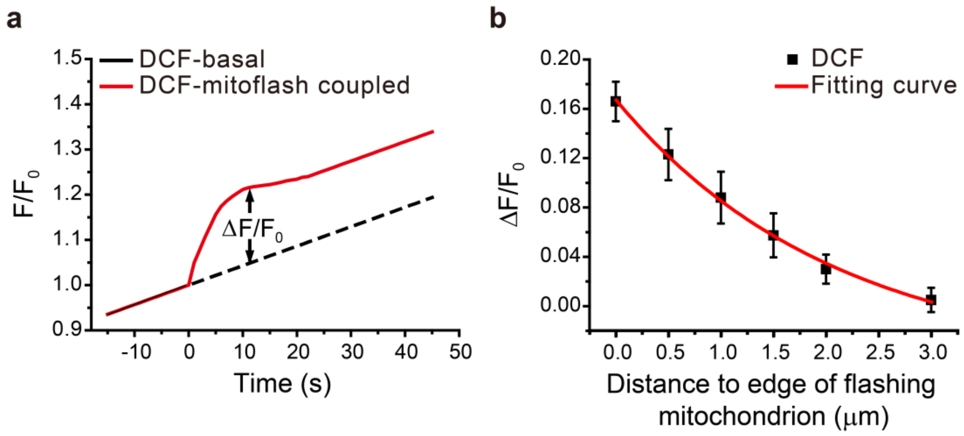

Supplementary Figure 8 Cytosolic ROS signals from flashing mitochondria.

(**a**) Schematic diagram showing DCF signals associated with mitoflashes, quantified as ($\Delta F/F_0 = DCF_{\text{coupled with mitoflash}} - DCF_{\text{baseline}}$). (**b**) Amplitude of local DCF signal (peak $\Delta F/F_0$) as a function of the distance from the near end of the corresponding mitoflash. A single-exponential fitting (smooth line) yielded a spatial decay constant of 2.07 μm (mean ± s.e.m.) (r^2=0.99).

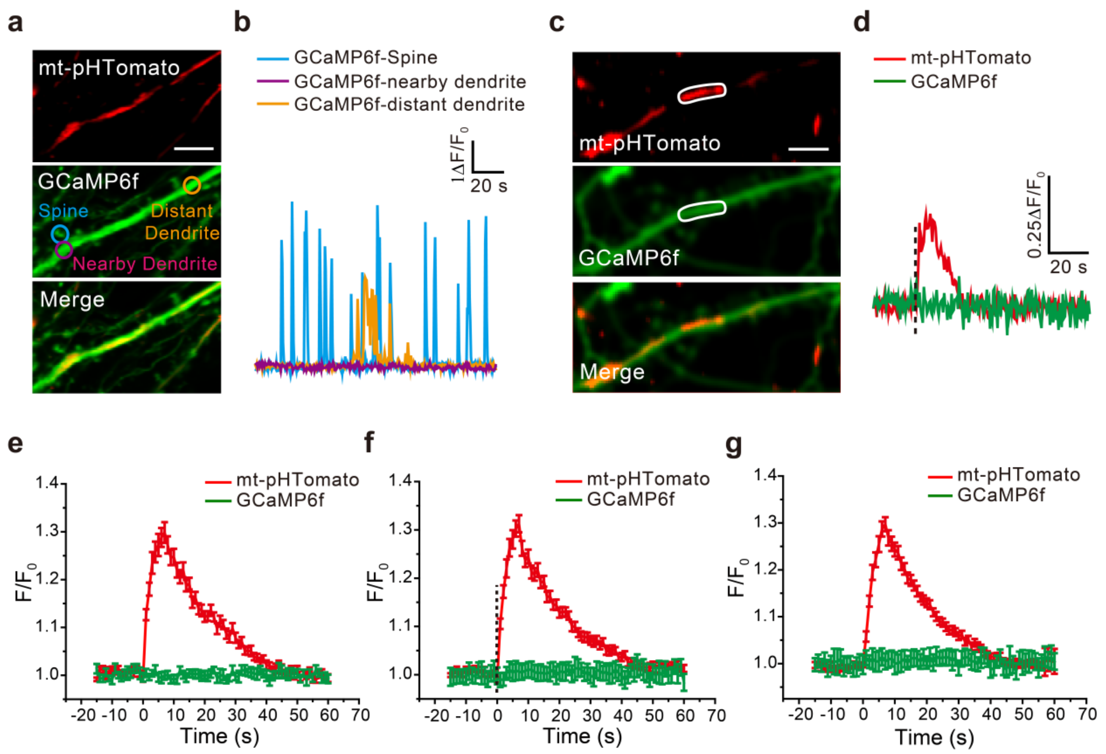

Supplementary Figure 9 Lack of local cytosolic calcium change associated with dendritic mitoflashes.

(**a**) Simultaneous imaging of dendritic mitoflashes (reported by mt-pHTomato) and cytosolic calcium (reported by GCaMP6f). Scale bar, 5μm. (**b**) Spontaneous spine and dendritic calcium transients reported by GCaMP6f. Traces were obtained from a single spine, its nearby dendritic area, and a distant dendritic area, as marked by circles in (**a**). (**c, d**) Representative example showing that a dendritic PA-mitoflash does not evoke any detectable change in local cytosolic calcium. White circles in (**c**) delimit a region of interest encompassing the target mitochondrion. Scale bar, 5μm. Dashed line in (**d**) indicates the time of laser stimulation. (**e-g**) Average time courses of local dendritic calcium changes during spontaneous (**e**), photo-activated (**f**), and nigericin-induced (**g**) mitoflashes (mean±s.e.m.). n= 88, 76, and 131mitoflashes, respectively.

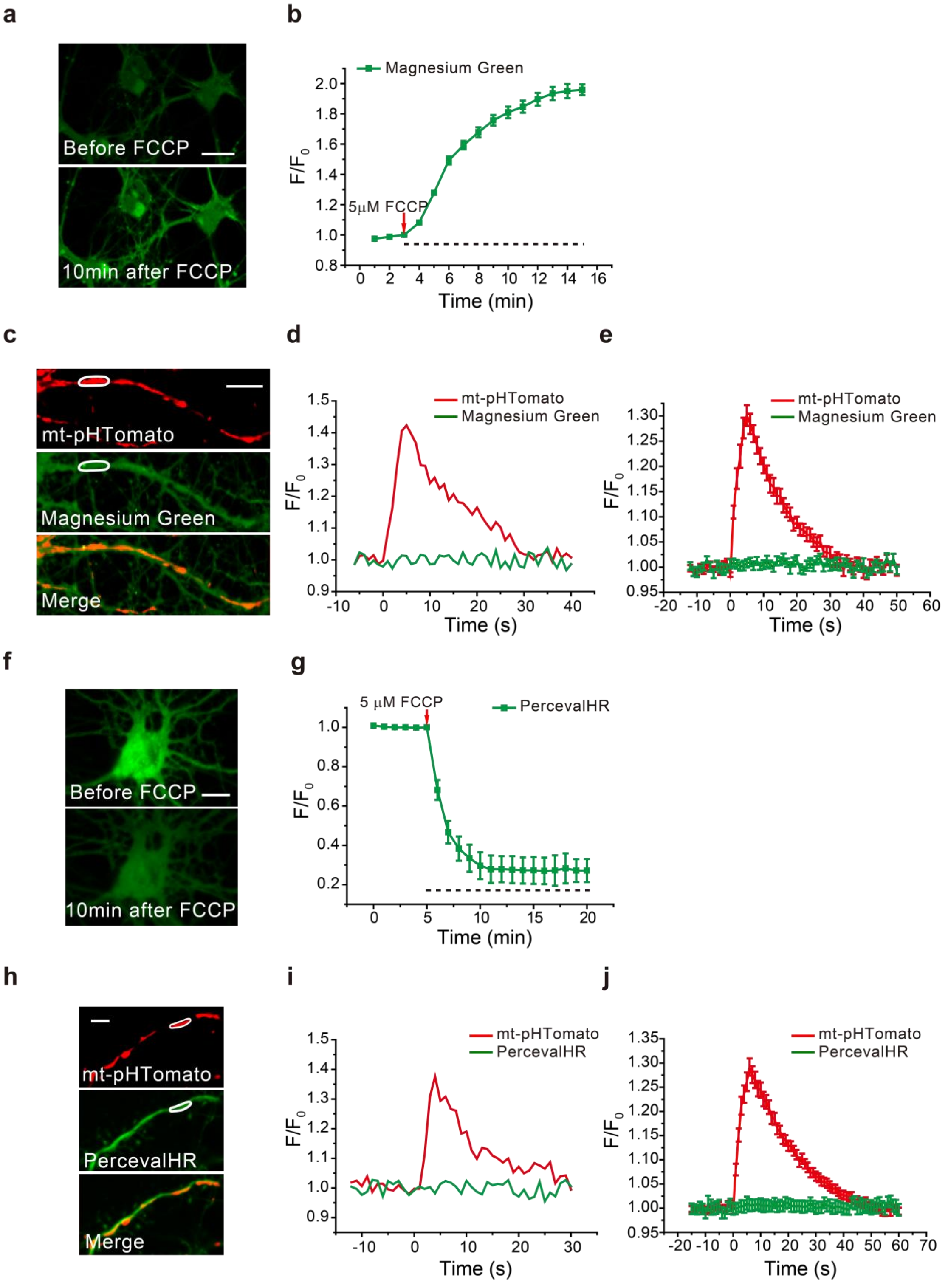

Supplementary Figure 10 Spontaneous mitoflash is not accompanied by any changes in local cytosolic ATP concentration.

(**a**) Representative images of magnesium green fluorescence in neurons before and after FCCP (5 μm)-induced ATP depletion. Scale bar, 10 μm. (**b**) Time course of magnesium green signal change during FCCP stimulation. An increase in magnesium

green fluorescence reflects a decrease in ATP concentration, the latter gives rise to an increase in free magnesium concentration (mean±s.e.m.). n=33 neurons from 2 batches. (**c**) Representative images showing dendritic mitochondria and cytosolic magnesium green signal. Scale bar, 5μm. (**d**) Time courses of a spontaneous mitoflash reported by mt-pHTomato (circled in **c**) and its companion magnesium green signal. (**e**) Statistics. n=117 events in 24 neurons from 3 batches. (**f**) Representative images of percevalHR before and after FCCP (5μm) induced ATP depletion in neurons (mean±s.e.m.). Scale bar, 10μm. (**g**) Time course of percevalHR signal change during FCCP stimulation. Decrease of percevalHR signal (488nm excitation) indicates decrease of cytosolic ATP concentration (mean±s.e.m.). n=9 neurons from 3 batches. (**h**) Representative images of dendritic mitochondria and percevalHR signal. Scale bar, 5μm. (**i**) Time courses of a mt-pHTomato detected mitoflash (circled in **h**) and accompanying cytosolic percevalHR signal. (**j**) Statistics (mean±s.e.m.). n=84 mitoflashes in 12 neuons from 3 batches.

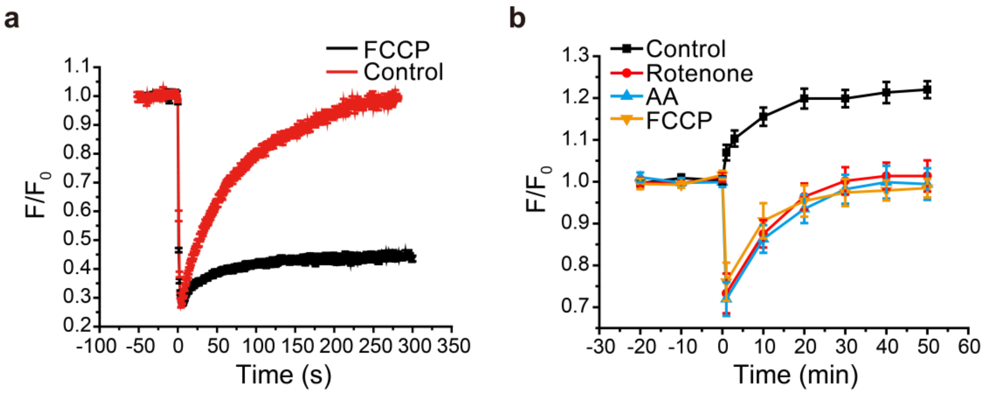

Supplementary Figure 11 ETC inhibitors and the oxidative phosphorylation uncoupler disrupt spine actin dynamics and abolish spine sLTP.

(**a**) Fluorescence recovery of actin-mcherry after photobleaching (FRAP) shows spine actin dynamics with and without FCCP treatment (20min, 300nM) (mean ±s.e.m.). (**b**) Time-dependent spine morphology changes in response to LS glutamate uncaging stimulation with and without ETC and oxidative phosphorylation inhibitors (mean ±s.e.m.).

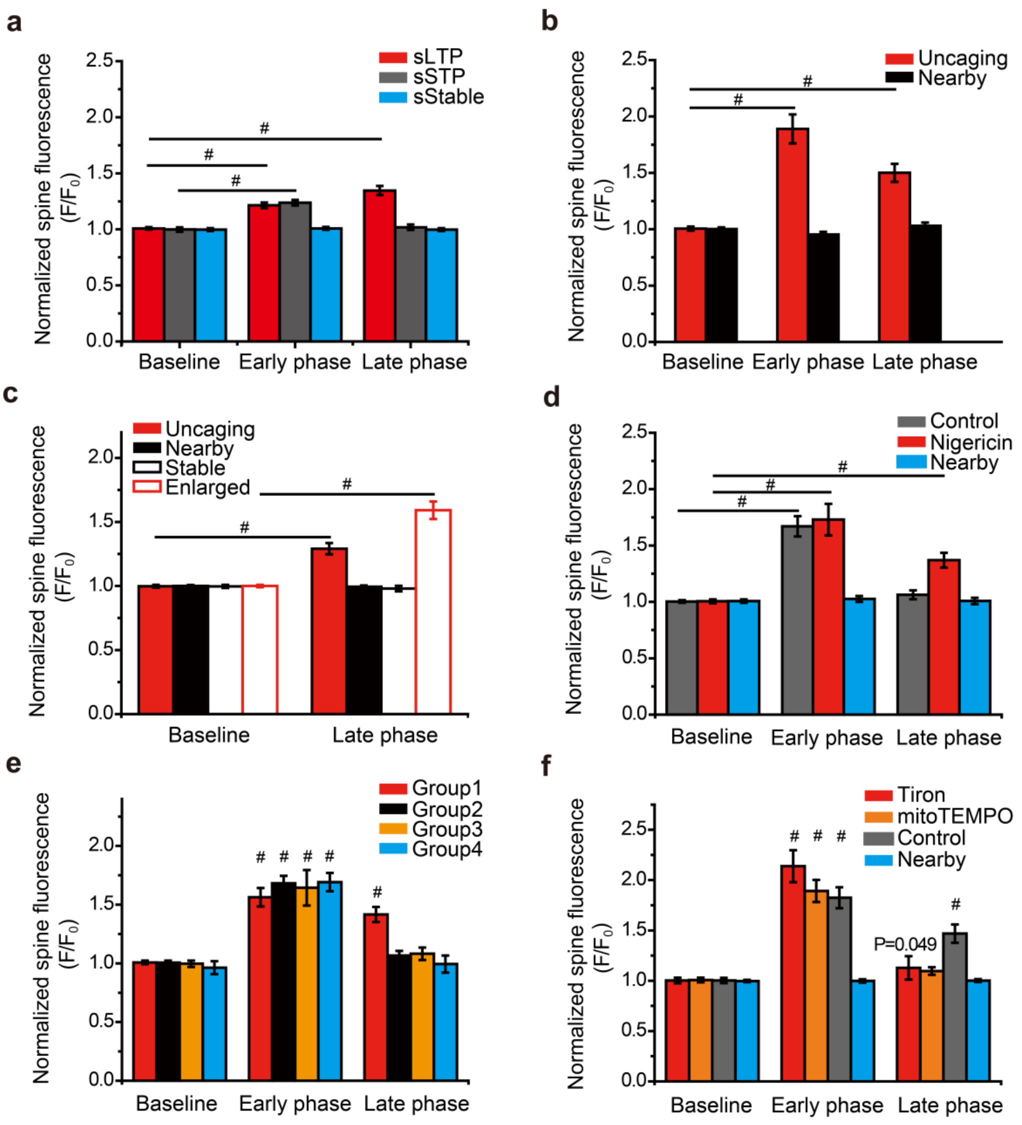

Supplementary Figure 12 Statistics of spine size changes as in Figures 1, 2, 4, 5.

(**a**) Size changes after HFS as in (Figure **1h**). The baseline is the averaged normalized spine fluorescence before HFS. The early phase is the averaged spine fluorescence (normalized) at 1 min and 3 min. The late phase is the averaged spine fluorescence (normalized) 30 min after HFS. n = 8 neurons from 4 batches. (**b**) Size changes after HS-glutamate uncaging as in (Figure **2d**). The baseline is the averaged normalized spine fluorescence before HS-glutamate uncaging. The early phase is the averaged spine fluorescence (normalized) at 1 min and 3 min. The late phase is the averaged spine fluorescence (normalized) 30 min after glutamate uncaging. (**c**) Long-term size changes after LS-glutamate uncaging as in (Figure **2g**). (**d**) Size changes after HW-glutamate uncaging as in (Figure **4b**). (**e**) Size changes after HW-glutamate uncaging as in (Figure **4f**). (**f**) Size changes after HS-glutamate uncaging as in (Figure **5d**). Error bars are SEM; #P <0.001 (paired sample t-test, early/late phase vs baseline).

Supplementary Table

Supplementary Table 1 Parameters of dendritic mitoflashes under different conditions.

**Mitoflash genesis**	**Time to peak**	**$\Delta F/F_0$**	**FDHM**
Spontaneous	7.61 ± 0.45 s	0.47 ± 0.02	12.96 ± 0.57 s
Photoactivated	6.78 ± 0.27 s	0.49 ± 0.02	12.89 ± 0.47 s
Nigericin-induced	6.85 ± 0.20 s	0.48 ± 0.01	13.02 ± 0.34 s

Data are mean ± s.e.m.; 500–900 mitoflash events were analyzed for each condition. FDHM: full duration at half maximum.
